# Induction therapy with mesenchymal stromal cells in kidney transplantation: a meta-analysis

**DOI:** 10.1186/s13287-021-02219-7

**Published:** 2021-03-01

**Authors:** Lingfei Zhao, Chenxia Hu, Fei Han, Dajin Chen, Jun Cheng, Jianyong Wu, Wenhan Peng, Jianghua Chen

**Affiliations:** 1 Kidney Disease Center, The First Affiliated Hospital, College of Medicine, Zhejiang University, Hangzhou, People’s Republic of China; 2 Institute of Nephrology, Key Laboratory of Kidney Disease Prevention and Control Technology, Zhejiang University, Hangzhou, Zhejiang People’s Republic of China; 3 State Key Laboratory for Diagnosis and Treatment of Infectious Diseases, College of Medicine, The First Affiliated Hospital, Zhejiang University, Hangzhou, Zhejiang People’s Republic of China

**Keywords:** Mesenchymal stromal cells, Induction therapy, Kidney transplantation

## Abstract

**Objective:**

The aim of this meta-analysis was to evaluate the therapeutic effects of mesenchymal stromal cells (MSCs) versus traditional regimens for induction therapy in kidney transplantation (KT), especially the safety of MSC infusion, practicability of MSCs as induction therapy agents, and posttransplant complications.

**Methods:**

PubMed, Embase, EBSCO, Ovid, and the Cochrane Library were searched for prospective clinical trials that compared MSCs with traditional regimens for induction therapy in KT.

**Results:**

Four trials were included, including a total of 197 patients. The pooled results revealed that MSC therapy had a lower 1-year infection rate than did the traditional therapies (RR = 0.65, 95% CI: 0.46–0.9, *P* = 0.01). There were no significant differences between the two protocols regarding the 1-year acute rejection (AR) rate (RR = 0.77, 95% CI: 0.41–1.45, *P* = 0.42), 1-year graft survival rate (RR = 0.99, 95% CI: 0.95–1.03, *P* = 0.74), delayed graft function (DGF) rate (RR = 0.54, 95% CI: 0.21–1.38, *P* = 0.2) and renal graft function at 1 month (MD = −1.56, 95% CI: − 14.2–11.08, *p* = 0.81), 3 months (MD = 0.15, 95% CI: − 5.63–5.93, *p* = 0.96), 6 months (MD = − 1.95, 95% CI: − 9.87–5.97, *p* = 0.63), and 12 months (MD = − 1.13, 95% CI: − 7.16–4.89, *p* = 0.71) postsurgery. Subgroup analysis demonstrated that the 1-year AR rate, 1-year graft survival rate, DGF rate, and renal graft function at 12 months postsurgery did not significantly differ between the low-dose calcineurin inhibitor (CNI) group and the standard-dose CNI group, indicating the potential benefits of successful CNI sparing in combination with MSC treatment. Moreover, when MSCs were applied as an alternative therapy rather than an additional therapy or allogeneic MSCs were utilized instead of autologous MSCs, all of the outcomes mentioned above were comparable.

**Conclusion:**

Induction therapy with MSCs is safe and has similar immune response modulation effects to those of traditional regimens in the short term in KT recipients. However, regarding the long-term effects, as suggested by the 1-year infection rate and the potential of CNI sparing, MSC therapy has significant advantages. However, these advantages should be further verified in more well-designed, multicenter randomized controlled trials (RCTs) with large sample sizes and long follow-up periods.

**Supplementary Information:**

The online version contains supplementary material available at 10.1186/s13287-021-02219-7.

## Introduction

Kidney transplantation (KT) is still the best treatment choice for end-stage renal disease (ESRD). Due to the development of tissue type-matching and immunosuppressive agents, the risk for acute rejection (AR) has been effectively reduced. However, posttransplant complications related to current immunosuppressive drugs are new issues that should be resolved. The long-term consumption of regular immunosuppressive drugs, including corticosteroids, calcineurin inhibitors (CNIs), antimetabolites, and sometimes lymphodepletion, can significantly increase the risk of some important adverse effects, such as nephrotoxicity, infection, tumorigenicity, diabetes, and cardiovascular diseases, which can affect the long-term graft outcomes and can even sometimes be life-threatening [1–5]. Infectious complications are common following KT and rank among the top five causes of patient deaths with allograft function [6]. Despite great efforts in drug innovations, the drawbacks mentioned above have not yet been resolved, and the hazards affecting long-term graft survival have failed to substantially decrease [7]. Novel immunosuppressive strategies that minimize posttransplant complications while maintaining adequate immunosuppression need to be explored.

Induction therapy, maintenance therapy, and AR therapy are the three main major stages of immunosuppressive therapy in KT. Over the last 20 years, the rate of induction therapy being applied in KT has increased from less than 30% to greater than 80% [8]. The most important factor leading to this rapid increase is its ability to reduce the historically high risk of acute allograft loss [9]. Usually, induction therapy is initiated perioperatively and ended within 3–14 days after surgery, in accordance with the theory that the upregulation of inflammatory factors due to cold and warm ischemia injury leads to a high risk of AR during this period [10]. Wagner et al. demonstrated that the positive improvements in the early graft survival rate in past decades were partially associated with the use of induction therapy [11]. In addition to the benefit regarding short-term allograft loss, another important reason for the introduction of induction therapy in KT is to reduce the need for or avoid maintenance therapy. Several trials have verified that due to induction therapy, a part of the traditional triple immunosuppressive regimen could be spared without the rate of acute or chronic rejection increasing [12, 13]. Long-term immunosuppressive minimization is meaningful for the prevention of drug toxicity over the long term after surgery. As it not only affects short-term peri-operative immunologic factors but also sets in motion a cascade of events lasting for a long period, induction therapy without a doubt should play an important role in the exploration of novel immunosuppressive strategies.

Traditionally, induction therapy can be categorized into a T cell depleting strategy and a T cell nondepleting strategy. The former contains antithymocyte globulin (ATG), anti-CD3 antibodies (OKT3), and alemtuzumab, while anti-IL-2 antibodies such as daclizumab, basiliximab, and anti-CD20 antibodies such as rituximab and cytoxan are regarded as T cell nondepleting agents. Currently, there is still a lack of consensus regarding the best induction therapy. Stem cell-based therapies are considered novel approaches that modulate immune responses during organ transplantation and have emerged over the last 10 years [14]. Among them, mesenchymal stromal cells (MSCs) have been proposed as promising candidates. MSCs are a type of cell that has the abilities of self-renewal, regeneration, proliferation, and three-lineage differentiation. Moreover, the limited expression of class II MHC molecules in its resting state makes it a low immunogenicity agent [15]. Functionally, by paracrine/endocrine actions such as the secretion of cytokines and growth factors, MSCs are able to interact with several key factors in both the innate and adaptive immune systems, playing immunoregulatory roles [16]. In other solid organ transplantation experimental models, studies have shown that MSCs have the potential to induce long-term graft acceptance when they are administered alone or in combination with short-term immunosuppressive treatments [17]. This evidence and that showing the clinical effectiveness of MSCs in the treatment of graft-versus-host disease (GVHD) motivated the use of MSCs as an induction therapy in KT [18]. Based on the functions of MSCs, the main reason MSCs were introduced as an induction therapy was to modulate the immune system after transplantation, which may help decrease the need for life-long immunosuppressive drugs and decrease the risk of posttransplant complications.

However, recent studies that used MSCs as induction therapy in KT have reported inconsistent results. Some studies have demonstrated that MSC treatment is effective in decreasing the ratio of memory/effector CD8(+) T cells, promoting renal functional recovery, or reducing the incidence of opportunistic infections [19–21]. However, others have suggested that MSCs are not advantageous over traditional regimens [22–24]. There even exists a study indicating that MSCs play a deleterious role regarding allograft survival [25]. Data on the risk of infections associated with the procedure are also inconsistent [21, 26]. Moreover, concern regarding the application of allogeneic MSCs when autologous MSCs are not suitable still exists, regardless of whether the MSCs are organ donor-derived or third party-derived. Whether MSCs are practicable for use in induction protocols is unclear. In this meta-analysis, we included all available clinical trials related to the application of MSCs as induction therapy in KT. We mainly focused on the safety and practicability of MSC infusion compared with traditional regimens as induction therapy in KT, especially in terms of the infusion reactions, AR rate, DGF rate, and allograft survival. Posttransplant complications, especially the risk of infections during the follow-up, were also closely considered. By summarizing these articles, we intended to provide an up-to-date view of this innovative induction regimen in KT, making it possible to minimize posttransplant complications without increasing the rate of rejection or hindering allograft survival, thereby leading to better prognoses for these patients.

## Materials and methods

### Search strategy

We searched PubMed, Embase, EBSCO, Ovid, and the Cochrane Library for related articles published after 1970. The last date a search was conducted was June 1, 2020. The search terms used were as follows: “mesenchymal stromal cells,” “mesenchymal stem cells,” “renal transplantation,” and “kidney transplantation.” The above terms and their combinations were also searched. All clinical trials that compared mesenchymal stromal cells with traditional regimens as induction therapy in KT were retrieved. There were no language restrictions for inclusion in this meta-analysis. The references within the included articles were also searched by hand. The abstracts of the articles were independently analyzed by two of the authors (L.F. Zhao and C.X. Hu) to determine whether the articles met the inclusion criteria. Disagreements between these two investigators were resolved by consensus.

### Inclusion criteria

We included all clinical trials that met all of the following criteria: (1) the study was a trial of adult KT; (2) the study compared the infusion of MSCs versus traditional regimens as induction therapy; (3) the baseline characteristics of the patients were matched between the two groups; (4) the trial assessed at least three of the following outcomes: the 1-year AR rate, 1-year infection rate, and renal graft function at 12 months postsurgery; and (5) the follow-up period lasted ≥1 year.

### Exclusion criteria

Studies enrolling pediatric patients were excluded. Studies enrolling patients with ABO blood incompatibility for KT were excluded.

### Data extraction

Data were extracted for all included trials by the two reviewers (L.F. Zhao and C.X. Hu) independently. Disagreements between these two reviewers were resolved by discussion. We extracted data from each study, including the first authors, year of publication, design of the trial, population characteristics, cases, duration of follow-up, interventions, MSC type, MSC source, MSC doses, and procedure and maintenance immunosuppressants.

### Outcomes of interest

The following reported outcomes were used to compare the therapeutic effects of MSCs with traditional regimens for induction therapy in KT: (1) 1-year AR rate, (2) 1-year graft survival rate, (3) 1-year infection rate, (4) delayed graft function (DGF) rate, and (5) renal graft function at 1, 3, 6, and 12 months postsurgery.

### Quality assessment

The quality of the RCTs, according to the following domains, was assessed using a modified Jadad scoring system: adequate randomization (2 = described in detail with proper methods of randomization, 1 = performed randomization, but no details were reported, 0 = did not perform randomization), allocation concealment (2 = described in detail with proper methods of allocation concealment, 1 = stated that allocation concealment was performed, but no details were provided, 0 = not performed properly), blinding (2 = double-blind, 1 = single-blind, 0 = open-label), completeness of the follow-up (1 = reported the number of patients excluded and the reasons for exclusion, 0 = not reported), and intention-to-treat (ITT) analysis. The maximum score was 7 points, and score higher than 4 points represented high-quality studies.

Moreover, the quality of the cohort studies was assessed using the Newcastle–Ottawa scale, which addressed the selection process (0 to 4 points), comparability (0 to 1 points), and outcomes (0 to 3 points). The maximum score was 8 points, with higher scores representing higher methodological quality.

### Statistical analysis

This meta-analysis was conducted in accordance with the Cochrane Collaboration meta-analysis guidelines [27]. Statistical analyses were performed using RevMan 5.1 statistical software (Cochrane Collaboration, Oxford, UK). The data were pooled using a fixed-effects model; if there was significant heterogeneity, the results were confirmed using a random-effects statistical model. For dichotomous outcomes, the results were expressed as risk ratios (RRs) with 95% confidence intervals (CIs). For continuous outcomes, we expressed the results using weighted mean differences (WMDs) with 95% CIs. We also assessed the heterogeneity of the results by performing the chi-square test and evaluated the extent of inconsistency using the *I*^2^ statistic. *I*^2^ values > 25%, > 50%, and > 75% were defined to indicate mild, moderate, and severe heterogeneity, respectively. *P* < 0.05 was considered statistically significant.

## Results

### Included studies

**Fig. 1:**
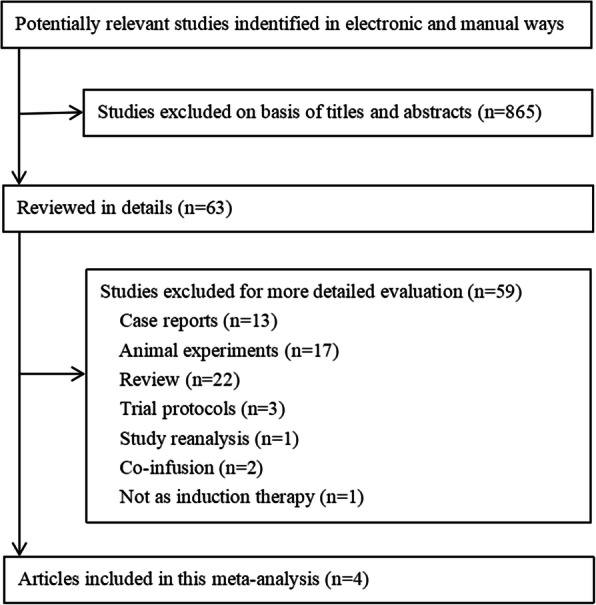
Flowchart of meta-analysis

The electronic and manual searches yielded 928 citations. A total of 865 citations were excluded after the titles and abstracts were read. Among the remaining 63 studies, there were 13 case reports, 17 animal experiments, 22 reviews, and three trial protocols. In addition, one study was a reanalysis of a former study, two studies reported the outcomes of co-fusion MSCs together with other stem cells, and one study did not intend to inject MSCs for induction therapy. Finally, four trials, conducted by the Erpicum group, Tan group, Sun group, and Pan group were included in this analysis [20–23]. Particularly, one cohort in the Tan group was not included, based on the methodology of a previous report [28]. So the total number of included patients in this meta-analysis was 197 (Fig. 1).

### Study characteristics

The details including the design of the trial, population characteristics, number of cases, duration of follow-up, interventions, MSC type, MSC source, MSC doses, and procedure and maintenance immunosuppressants are summarized in Table 1. Particularly, one trial assessed the application of MSCs as an alternative treatment to anti-IL-2 receptor antibodies (Tan group) [21], while the other three used MSCs as an additional comparator (Erpicum group, Sun group, and Pan group) [20, 22, 23]. Additionally, in the trial by Tan et al., autologous bone marrow-derived MSCs (BM-MSCs) were administered. Specifically, bone marrow cells were aspirated from the kidney recipient 1 month before the transplant and were then isolated and expanded [21]. In the remaining three trials, allogeneic MSCs were infused. In details, Erpicum et al. and Pan et al. tried to infuse BM-MSCs [20, 23], while umbilical cord-derived MSCs (UC-MSCs) were administered by Sun et al. in the remaining trial [22]. The Tan group and Pan group tried to reduce the doses of CNIs during the maintenance period, with an approximately 20% reduction in the Tan group [21] and an approximately 40% decrease in the Pan group [23]. Three trials used two injections of transplanted MSCs, while the remaining trial conducted by the Erpicum group used a one-injection regimen [20]. The specific timepoint of the MSC intervention also varied across trials. The patients in the Erpicum group received MSC treatment on D3 ± 2 with a dose of approximately 1.5 × 10^6^–3 × 10^6^ cells/kg [20]. The Sun group separately infused 2 × 10^6^ cells/kg and 5 × 10^6^ cells 30 min before surgery and during surgery [22], while the Pan group chose to inject 5 × 10^6^ MSCs during surgery, followed by 2 × 10^6^ cells/kg on D30 [23]. The Tan group transplanted two doses of 1–2 × 10^6^ cells/kg MSCs before surgery and on D14 postsurgery [21]. No infusion-related adverse effects were observed in any of these four trials [20–23]. Except for the study by Erpicum et al. and Sun et al. [20, 22], the remaining two trials included living-related donor kidney transplant recipients. The quality assessment results are shown in Table 2. All four included trials were regarded as high quality.

**Table 1:** Characteristics of included studies

Author	Year	Design of the study	Population characteristics	Cases		Follow up (month)	Interventions		MSCs types	MSCs sources	MSCs doses and procedure	Maintenance immunosuppressants	
				Treatment group	Control group		Treatment group	Control group				Treatment group	Control group
Erpicum [20]	2018	Single-centre, nonrandomized, controlled study	Deceased donor kidney transplant recipients	10	10	12 months	MSC + Anti–IL-2 receptor antibody (D0 + D4)	Anti–IL-2 receptor antibody (D0 + D4)	Allogeneic	BM	One intravenous injection (1.5 × 10^6^–3 × 10^6^ cells/kg D3 ± 2)	Steroids, MMF, CNIs	Steroids, MMF, CNIs
Tan [21]	2012	Single-centre, prospective RCT	Living-related donor kidney transplant recipients	52	51	12 months	MSCs	Anti–IL-2 receptor antibody (20 mg D0 + D4)	Autologous	BM	Two intravenous injections (1–2 × 10^6^ cells/kg before surgery and D14)	Steroids, MMF, 80% of the standard of CNIs	Steroids, MMF, CNIs
Sun [22]	2018	Multi-center prospective RCT	Deceased donor kidney transplant recipients	21	21	12 months	MSC + ATG (50 mg/day D0–2)	ATG (50 mg/day D0–2)	Allogeneic	UC	Two intravenous injections (2 × 10^6^ cells/kg 30 mins before surgery, 5 × 10^6^ cells during surgery)	Steroids, MMF, CNIs	Steroids, MMF, CNIs
Pan [23]	2016	Single-centre, prospective, nonrandomized pilot study	Living-related donor kidney transplant recipients	16	16	24 months	MSCs+Cytoxan (200 mg/day D0–3)	Cytoxan (200 mg/day D0–3)	Allogeneic	BM	Two injections (5 × 10^6^ cells through renal allograft artery during surgery, 2 × 10^6^ cells/kg intravenously D30)	Steroids, MMF, 60% of the standard of CNIs	Steroids, MMF, CNIs

*RCT* Randomized control trial, *MSCs* Mesenchymal stromal cells, *ATG* Antithymocyte globulin, *BM* Bone marrow, *UC* Umbilical cord, *CNIs* Calcineurin inhibitors, *MMF* Mycophenolate mofetil

**Table 2:** Quality assessment of included studies

Author	Randomized adequately	Allocation concealment	Blinding	Completeness of follow-up	ITT analysis	Groups similar at baseline	Specific inclusion criteria	Modified Jadad score	Quality
Tan [21]	2	0	1	1	no	yes	yes	4	high
Sun [22]	2	0	1	1	no	yes	yes	4	high
Author	Representativeness of the exposed cohort	Selection of the nonexposed cohort	Ascertainment of exposure	Demonstration that outcome of interest was not present at start of study	Comparability of cohorts on the basis of the design or analysis	Assessment of outcome	Was follow-up long enough for outcomes to occur	Adequacy of follow up of cohorts	Quality
Erpicum [20]	1	1	1	1	1	1	1	1	high
Pan [23]	1	1	1	1	1	1	1	1	high

*ITT* Intention to treat

### Meta-analysis of MSCs versus traditional regimens as induction therapy (summarized in Table 3)

#### Effect on the 1-year AR rate

**Fig. 2:**
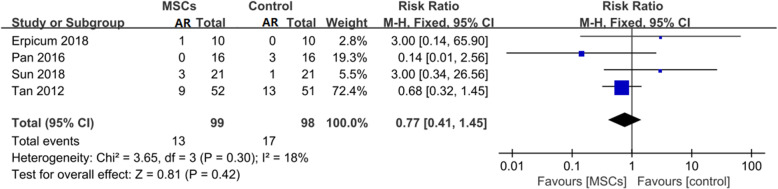
Effect on 1-year AR rate

All four trials studied the effect of MSC therapy on the 1-year AR rate in a total of 197 patients [20–23]. In these trials, 99 patients were assigned to the MSC group, and 98 patients were assigned to the control group. An analysis of the treatment effect on the 1-year AR rate is shown in Fig. 2. The forest plots display the results of the meta-analysis for the entire group (RR = 0.77, 95% CI: 0.41–1.45, *P* = 0.42) (Fig. 2). The results show that there were no significant differences in the 1-year AR rate between the two groups.

**Table 3:** Meta-analysis of MSCs versus traditional regimens as induction therapy

Outcomes	Number of trials	Number of patients	RR/WMD	95% CI	*p* value	Heterogeneity *p* value (%)
1-year AR rate	4	197	0.77	0.41,1.45	0.42	18
1-year graft survival rate	3	160	0.99	0.93,1.05	0.69	0
1-year infection rate	4	197	0.65	0.46,0.9	0.01	3
DGF rate	2	145	0.54	0.21,1.38	0.2	34
Renal graft function postsurgery						
1 month	3	155	-1.56	−14.2, 11.08	0.81	72
3 month	3	155	0.15	−5.63,5.93	0.96	0
6 month	3	155	−1.95	−9.87,5.97	0.63	62
12 month	4	197	−1.13	−7.16,4.89	0.71	0

*MSCs* mesenchymal stromal cells, *AR* acute rejection, *DGF* delay graft function, *RR* risk ratio, *WMD* weighted mean difference, *CI* confidence intervals

#### Effect on the 1-year graft survival rate

Three trials assessed the effect on the 1-year graft survival rate in a total of 160 patients [20–22]. Eighty patients were assigned to the MSC group, and 80 patients were assigned to the control group.

**Fig. 3:**
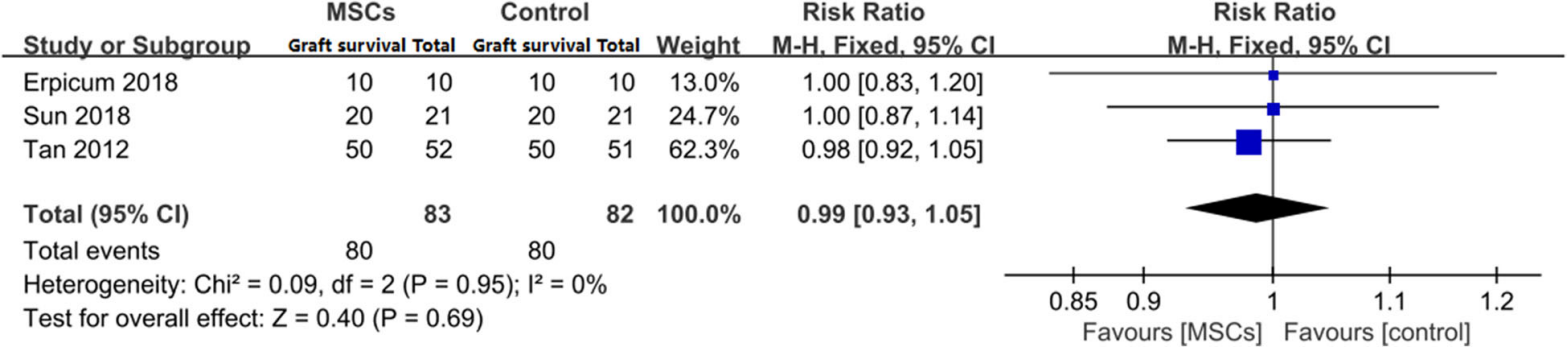
Effect on 1-year graft survival rate

An analysis of the treatment effect on the 1-year graft survival rate is shown in Fig. 3. The forest plots display the results of the meta-analysis for the entire group (RR = 0.99, 95% CI: 0.93–1.05, *P* = 0.69). Our meta-analysis indicated that treatment with MSCs induces a 1-year graft survival rate comparable with that of the control group.

#### Effect on the 1-year infection rate

**Fig. 4:**
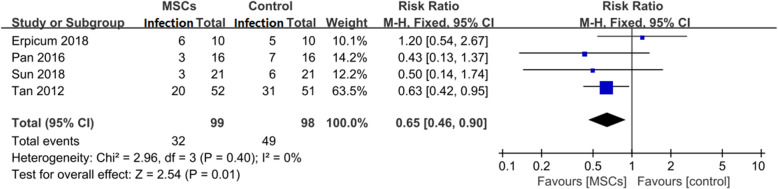
Effect on 1-year infection rate

All four included trials reported the 1-year infection rate for a total of 197 patients [20–23]. As shown in Fig. 4, 99 patients were assigned to the MSC group, and 98 patients were assigned to the control group. At the 1-year follow-up, the infection rate in the MSC group was significantly lower than that in the control group (RR = 0.65, 95% CI: 0.46–0.9, *P* = 0.01). This evidence suggests that induction therapy with MSCs can effectively reduce the infection rate after KT.

#### Effect on the DGF rate

**Fig. 5:**
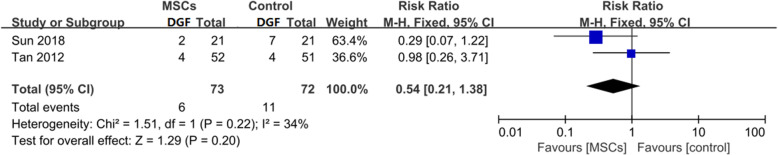
Effect on DGF rate

Two of the four trials, involving 145 patients, investigated the effect of MSC therapy on the DGF rate in adult KT patients [21, 22]. Seventy-three patients were assigned to the MSC group, and 72 patients were assigned to the control group (Fig. 5).

An analysis of the effect of treatment on the DGF rate is shown in Fig. 5. The forest plots display the results of the meta-analysis for the entire group (RR = 0.54, 95% CI: 0.21–1.38, *P* = 0.2). According to our meta-analysis in which the weight of each study was taken into account, there were no significant differences in the DGF rate between the two groups.

#### Effect on renal graft function postsurgery

**Fig. 6:**
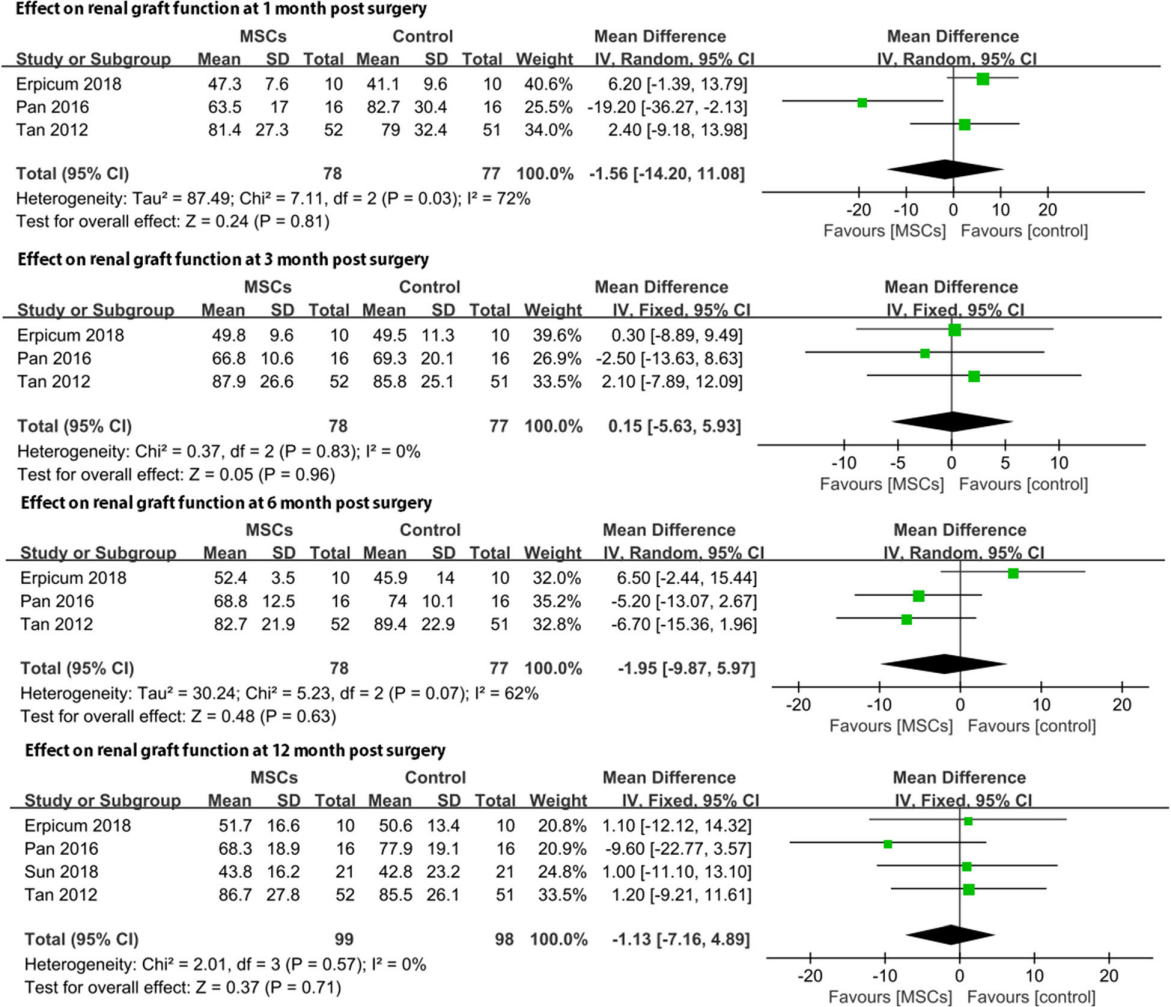
Effect on renal graft function at 1, 3, 6, and 12 months postsurgery

Renal graft function postsurgery was assessed at 1, 3, 6, and 12 months postsurgery. At every evaluation point, renal graft function was comparable between the two groups (Fig. 6), including (MD = −1.56, 95% CI: − 14.2–11.08, *p* = 0.81) at 1 month, (MD = 0.15, 95% CI: − 5.63–5.93, *p* = 0.96) at 3 months (MD = −1.41, 95% CI: − 5.69–2.87, *p* = 0.52) at 6 months, and (MD = −1.13, 95% CI: − 7.16–4.89, *p* = 0.71) at 12 months. Our meta-analysis demonstrated that the therapeutic effects on renal graft function were the same in the MSC group and control group.

### Sensitivity analysis

There were some differences in the designs of the included trials, mainly in the maintenance immunosuppressants used, whether MSCs were used as an alternative or additional therapy and the MSC types. Thus, subgroup analysis was conducted to explore the level of homogeneity. According to the subgroup analysis, the level of homogeneity was acceptable in this meta-analysis.

#### Subgroup analysis between the low-dose CNI group and the standard-dose CNI group

To evaluate whether MSCs can induce successful CNI sparing, we conducted a subgroup analysis. The results are summarized in Table 4 (Supplementary Figures 1, 2, 3, 4, 5). Subgroup analysis demonstrated that the 1-year AR rate, 1-year graft survival rate, DGF rate, 1-year infection rate, and renal graft function at 12 months postsurgery did not significantly differ between the low-dose CNI group and the standard-dose CNI group, indicating the potential benefits of successful CNI sparing in combination with MSC treatment.

**Table 4:** Subgroup analysis between the low-dose CNI group and the standard-dose CNI group

Outcomes	Low-dose CNI				Standard-dose CNI				Subgroup differences	
	Number of trials	Number of patients	RR/WMD	95% CI	Number of trials	Number of patients	RR/WMD	95% CI	*p* value	Heterogeneity *p* value (%)
1-year AR rate	2	135	0.57	0.27, 1.17	2	62	3	0.51, 17.82	0.09	65.4
1-year graft survival rate	1	103	0.98	0.92, 1.05	2	62	1	0.9, 1.11	0.77	0
DGF rate	1	103	0.98	0.26, 3.71	1	42	0.29	0.07, 1.22	0.22	33.7
1-year infection rate	2	135	0.6	0.4, 0.88	2	62	0.82	0.41, 1.63	0.43	0
Renal graft function postsurgery at 12 months	2	135	−2.96	−11.12, 5.21	2	62	1.05	−7.88, 9.97	0.52	0

*MSCs* mesenchymal stromal cells, *AR* acute rejection, *DGF* delay graft function, *RR* risk ratio, *WMD* weighted mean difference, *CI* confidence intervals

#### Subgroup analysis between the autologous MSC group and the allogeneic MSC group

There is still concern about the application of allogeneic MSCs in KT. Subgroup analysis was conducted. The results demonstrated that allogeneic MSCs did not affect the 1-year AR rate, 1-year graft survival rate, DGF rate, 1-year infection rate, or renal graft function at 12 months postsurgery. The results are summarized in Table 5 (Supplementary Figures 6, 7, 8, 9, 10).

**Table 5:** Subgroup analysis between the autologous/MSCs alternative therapy group and the allogeneic/MSCs additional therapy group

Outcomes	Autologous/MSCs alternative therapy				Allogeneic/MSCs additional therapy				Subgroup differences	
	Number of trials	Number of patients	RR/WMD	95% CI	Number of trials	Number of patients	RR/WMD	95% CI	*p* value	Heterogeneity *p* value (%)
1-year AR rate	1	103	0.68	0.32, 1.45	3	94	1	0.3, 3.32	0.59	0
1-year graft survival rate	1	103	0.98	0.92, 1.05	2	62	1	0.9, 1.11	0.77	0
DGF rate	1	103	0.98	0.26, 3.71	1	42	0.29	0.07, 1.22	0.22	33.7
1-year infection rate	1	103	0.63	0.42, 0.95	3	94	0.67	0.37, 1.2	0.89	0
Renal graft function postsurgery at 12 months	1	103	1.2	−9.21,11.61	3	94	−2.31	−9.7,5.08	0.59	0

*MSCs* mesenchymal stromal cells, *AR* acute rejection, *DGF* delay graft function, *RR* risk ratio, *WMD* weighted mean difference, *CI* confidence intervals

#### Subgroup analysis between the MSC alternative therapy group and the MSC additional therapy group

We also considered the impact of MSC alternative therapy on the 1-year AR rate, 1-year graft survival rate, DGF rate, 1-year infection rate, and renal graft function at 12 months postsurgery. The group that used MSCs as an alternative therapy was the same group that used autologous MSCs. Moreover, the data in the MSC additional therapy group and those in the allogeneic MSC group were similar. Therefore, the results of this subgroup analysis are similar to those of the comparison of the autologous and allogeneic MSC subgroups. According to the subgroup analysis, all of the outcomes mentioned above were comparable between the MSC alternative therapy group and the MSC additional therapy group. The results are summarized in Table 5 (Supplementary Figures 6, 7, 8, 9, 10).

## Discussion

To our knowledge, this is the first meta-analysis on the use of MSC therapy as an induction treatment in KT. The results from our meta-analysis demonstrate (1) that MSC infusion is a safe induction therapy; (2) this therapy is practicable, which means that this regimen is at least not inferior to traditional induction therapies in terms of AR events, the DGF rate, and real graft function postsurgery, regardless of whether it is used as an alternative regimen or an additional regimen; and (3) this therapy has advantages over traditional induction regimens in terms of a lower risk of infections during the follow-up and the potential for CNI sparing.

First, the infusion of MSCs as an induction therapy is safe. It was reported that despite the low expression of HLA molecules in the resting state and the inherent immunosuppressive properties, the injection of allogeneic MSCs is still associated with a risk of active recipients’ immune responses and induces the development of donor-specific antibodies [29, 30]. Embolism has also been reported in some cases [31]. In our meta-analysis, during the period of MSC infusion, no related adverse effects occurred in any of the four included trials, indicating that MSC infusion was tolerated.

Second, the infusion of MSCs as an induction therapy is practical. There still exists a concern that the infusion of MSCs will cause damage to the grafted kidney based on the undesirable results reported by Perico et al. in 2011. Perico et al. were the pioneers to attempt the application of MSCs in patients undergoing KT. However, both patients receiving MSC treatment in their study experienced transient serum creatinine increases from 7 to 14 days after MSC infusion, dampening our expectations of this regimen [25]. According to our meta-analysis, a major difference between the abovementioned study conducted by Perico et al. and those studies included in our research was that the timepoint of MSC infusion differed. Perico et al. tried to inject MSCs 7 days after surgery rather than pre- or peri-transplant infusion. After surgery, the infused MSCs followed the graft-originated inflammatory stimulus into the kidney, turning into pro-inflammatory-type cells rather than immunoregulatory-type cells and localizing in the lymphoid organs when the patient was injected before transplantation [32]. One of the patients in that study underwent graft biopsy and presented with focal interstitial inflammatory cell infiltration and deposition of complement C3 without signs of AR, resembling engraftment syndrome, which is commonly seen in bone marrow transplantation patients, verifying this explanation [33]. In our meta-analysis, in all four included trials, the first dose of MSCs was injected in the pre- or peritransplant period. Compared with standard induction regimens, the utilization of MSCs as an induction therapy did not increase the risk of AR or DGF (Figs. 2 and 5). Moreover, the 1-year graft survival rate and renal graft function at 1, 3, 6, and 12 months postsurgery were comparable between these two groups (Figs. 3 and 6). This evidence suggests that MSCs are practicable and not associated with poorer graft outcomes than are traditional regimens in the short term. MSCs could be an alternative choice for induction treatment in KT patients.

Third, but most importantly, our meta-analysis demonstrated a lower infection incidence in the MSC group. The RR for the 1-year infection rate was 0.73 0.65 (95% CI: 0.46–0.9, *P* = 0.01) in the MSC group compared with traditional regimens group (Fig. 4). One major concern for the application of MSCs in KT patients was whether such an expensive therapy was suitable to be used to merely prevent AR, an event that can be well controlled by conventional immunosuppressive drugs. The results from our research demonstrated another meaningful advantage of this therapy. The attention of the transplant community has turned from the inhibition of rejection reactions to long-term event-free survival [16]. Infection is an important part of this goal. In contrast with a study with a small sample size conducted by Reinders et al. that demonstrated a high opportunistic viral infection risk [26], our data provide evidence that patients who received MSC infusion develop fewer infections than do those in the control group. The different immune states of patients might account for this contradiction, as all the patients in Reinders’s study had signs of rejection or interstitial fibrosis/tubular atrophy (IF/TA) before MSC infusion.

Finally, some hints from the subgroup analysis deserve to be mentioned. Although the sample size was small and the analyses may be underpowered, the results of the subgroup analysis still provide useful information for future applications. (1) The subgroup analysis between the low-dose CNI group and the standard-dose CNI group suggested that the strategy with MSCs may successfully reduce the total dosage of CNIs required during the maintenance period. The lifelong intake of immunosuppressive drugs that are necessary to circumvent graft rejection inevitably lead to a high risk of morbidity and mortality in KT recipients. The idea to use of MSCs as an induction therapy was conceived to minimize immune suppression, especially during the maintenance period. Animal experiments showed that combination therapy with MSCs contributed to a subtherapeutic dose of rapamycin in promoting graft tolerance [34]. The subgroup analysis in our meta-analysis also revealed that the 1-year AR rate, 1-year graft survival rate, DGF rate, and renal graft function at 12 months postsurgery did not differ between the low-dose CNI group and the standard-dose CNI group (Supplementary Figures 1, 2, 3, 4, 5). In the study by Pan et al., the decreased CNI dosage did not affect graft survival over the 2 years of follow-up [23]. Although long-term data are still lacking, our meta-analysis suggests that successful CNI sparing can be achieved with MSC treatment. (2) The subgroup analysis between the autologous MSC group and the allogeneic MSC group suggested that allogeneic MSCs are as safe and practicable as autologous MSCs for induction therapy in KT. The need for the difficult, expensive, and time-consuming production of autologous MSCs, which takes several weeks to months, could be the major obstacle preventing their widespread use in clinical applications. The recipient’s health condition could also impact the function of infused cells [35]. Moreover, autologous MSCs were not suitable in the deceased-donor KT program, which was the primary source from which kidney grafts were retrieved. However, despite its low immunogenicity, concern about recipient sensitization was of special relevance when allogeneic MSCs were utilized. Our analysis might provide a degree of answer to this, but still remains a matter of debatable question whether to treat KT recipients with autologous MSCs or allogeneic ones. The advantages of applying allogeneic MSCs, especially third party-derived MSCs from large-scale clinical manufacturing, were the standardized production and preservation conditions. With these procedures, the variations in the quality and efficacy of MSCs could be controllable prior to infusion. Meanwhile, a reduction in the potential discrepancies in results across clinical studies could be another strength of this “off-the-shelf” cell product [16]. (3) Subgroup analysis between the MSC alternative therapy group and the MSC additional therapy group indicated that MSCs not only can be used as an additional therapy but also can be used to replace traditional induction therapies, such as anti–IL-2 receptor antibodies.

Despite the promising future, some limitations in our meta-analysis should also be mentioned. First, the sample size of our meta-analysis is not large enough. Only four trials containing a total of 197 patients were available in the meta-analysis. When handling the Tan group, one cohort receiving MSC-treated standard CNI dose regimen was excluded because of methodological restriction. It was a more reasonable way to combine the two MSC-treated CNI dose-cohorts into a single “treatment group” of 105 subjects. However, available methodology of meta-analysis could not provide such a way to combine related data in these two cohorts. We also tried to contact with the author to seek for the original data. However, we could not get a reply from them. So, we decided to exclude the MSC-treated standard CNI dose cohort. Previous study also did the same analysis when facing the same issue [28]. Besides the conclusions did not change when either MSC-treated standard CNI dose cohort or MSC-treated low CNI dose cohort was included (data not shown). In addition, most of the patients included in our meta-analysis were young KT recipients (average age in the included studies: Erpicum group 56.7 years, Tan group 38 years, Sun group 43.9 years, Pan group 29.97 years), which means that they have a low risk of AR and other major posttransplant complications. The conclusions of this meta-analysis should be interpreted with caution in higher risk recipients. Second, the follow-up time was not long enough. Most patients in our study were followed up for 1 year. This follow-up time is insufficient to reach a strong conclusion. Third, the quality of the included studies was assessed. Although all of the included studies met a criterion for high quality, they have some issues in regard to quality. For example, drawbacks in allocation concealment, blinding, and ITT analysis were identified in the studies by the Tan group [21] and Sun group [22]. Fourth, most of the included trials used BM-MSCs. The trial by Sun et al. used UC-MSCs [22]. MSCs derived from tissues such as adipose, amnion, and placenta tissues were not included in this meta-analysis. Additional trials with various kinds of MSCs should be conducted because MSCs from different sources differ in cytokine production, chemokine production, and immunomodulatory properties, especially between the bone marrow and adipose tissue [36, 37]. Fifth, the quality control of the MSCs was not mentioned in the original studies. Last but not least, the risk of tumor formation was not analyzed in our research or in the original articles. The concern for the development of tumors is a major barrier to translation into clinical settings. Although no studies to date have reported the de novo formation of tumors after MSC infusion in humans, this phenomenon should always be monitored. To overcome these limitations, more well-designed, multicenter RCTs with large sample sizes and long follow-up periods need to be conducted to further investigate this issue.

In conclusion, our meta-analysis demonstrated that MSC therapy is safe and practicable in inducing immune response modulation in recipients of KT with respect to traditional regimens. Some advantages of MSC therapy, such as a lower risk of infections and the potential of CNI sparing, were also revealed by this study. However, these findings were based on short-term follow-up data. Some clinical trials are ongoing and will eventually provide more evidence about the long-term risks and benefits of MSC therapy. We believe MSCs as an induction therapy in KT is promising and that more studies in this field should be conducted.

## Supplementary Information

**Additional file 1: Supplementary Figure 1.** Effect on 1-year AR rate between low-dose CNI group and the standard-dose CNI group.

**Additional file 2: Supplementary Figure 2.** Effect on 1-year graft survival rate between low-dose CNI group and the standard-dose CNI group.

**Additional file 3: Supplementary Figure 3.** Effect on 1-year infection between low-dose CNI group and the standard-dose CNI group.

**Additional file 4: Supplementary Figure 4.** Effect on DGF rate between low-dose CNI group and the standard-dose CNI group.

**Additional file 5: Supplementary Figure 5.** Effect on renal graft function at 12 months post surgery between low-dose CNI group and the standard-dose CNI group.

**Additional file 6: Supplementary Figure 6.** Effect on 1-year AR rate between autologous/MSC alternative therapy group and allogeneic/MSC additional therapy group.

**Additional file 7: Supplementary Figure 7.** Effect on 1-year graft survival rate between autologous/MSC alternative therapy group and allogeneic/MSC additional therapy group.

**Additional file 8: Supplementary Figure 8.** Effect on 1-year infection between autologous/MSC alternative therapy group and allogeneic/MSC additional therapy group.

**Additional file 9: Supplementary Figure 9.** Effect on DGF rate between autologous/MSC alternative therapy group and allogeneic/MSC additional therapy group.

**Additional file 10: Supplementary Figure 10.** Effect on renal graft function at 12 months post surgery between autologous/MSC alternative therapy group and allogeneic/MSC additional therapy group.

## Data Availability

Not applicable

## Abbreviations

MSCs: Mesenchymal stromal cells
KT: Kidney transplantation
AR: Acute rejection
DGF: Delayed graft function
CNI: Calcineurin inhibitor
RCTs: Randomized controlled trials
ESRD: End-stage renal disease
ATG: Antithymocyte globulin
OKT3: Anti-CD3 antibodies
GVHD: Graft-versus-host disease
ITT: Intention-to-treat
RRs: Risk ratios
CIs: Confidence intervals
WMDs: Weighted mean differences
BM-MSCs: Bone marrow-derived MSCs
UC-MSCs: Umbilical cord-derived MSCs
IF/TA: Interstitial fibrosis/tubular atrophy
MMF: Mycophenolate mofetil
