# Supplementary figures and images for: Induction therapy with mesenchymal stromal cells in kidney transplantation: a meta-analysis
Source: Stem Cell Res Ther. 2021 Mar 1;12:158. doi: 10.1186/s13287-021-02219-7 (PMC7923637; doi:10.1186/s13287-021-02219-7)

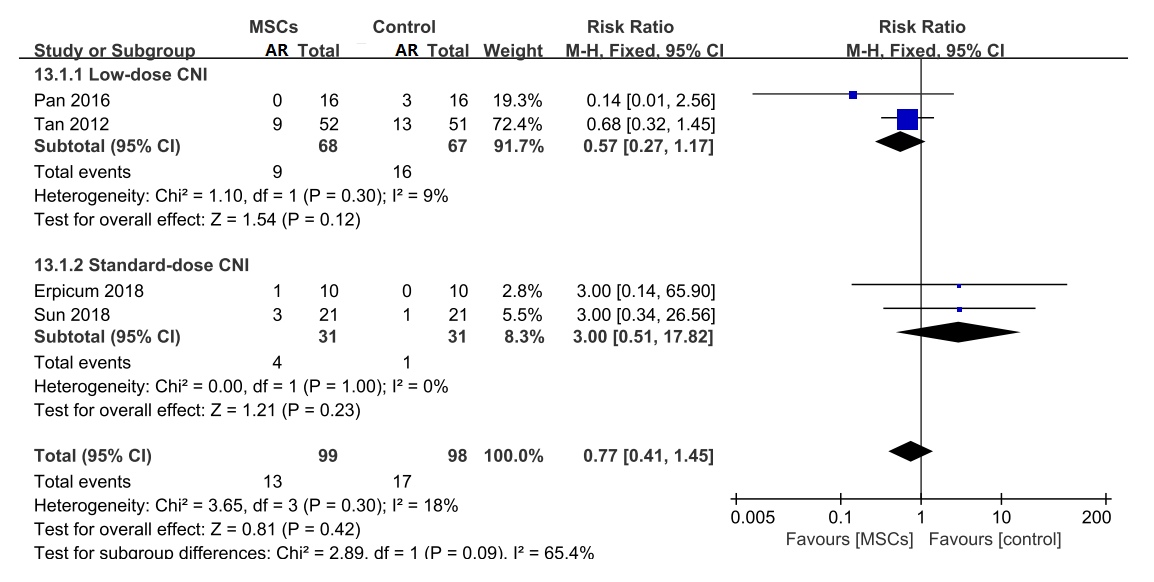

Supplement: Supplementary file 1 — Additional file 1: Supplementary Figure 1. Effect on 1-year AR rate between low-dose CNI group and the standard-dose CNI group. [file 13287_2021_2219_MOESM1_ESM.tif]

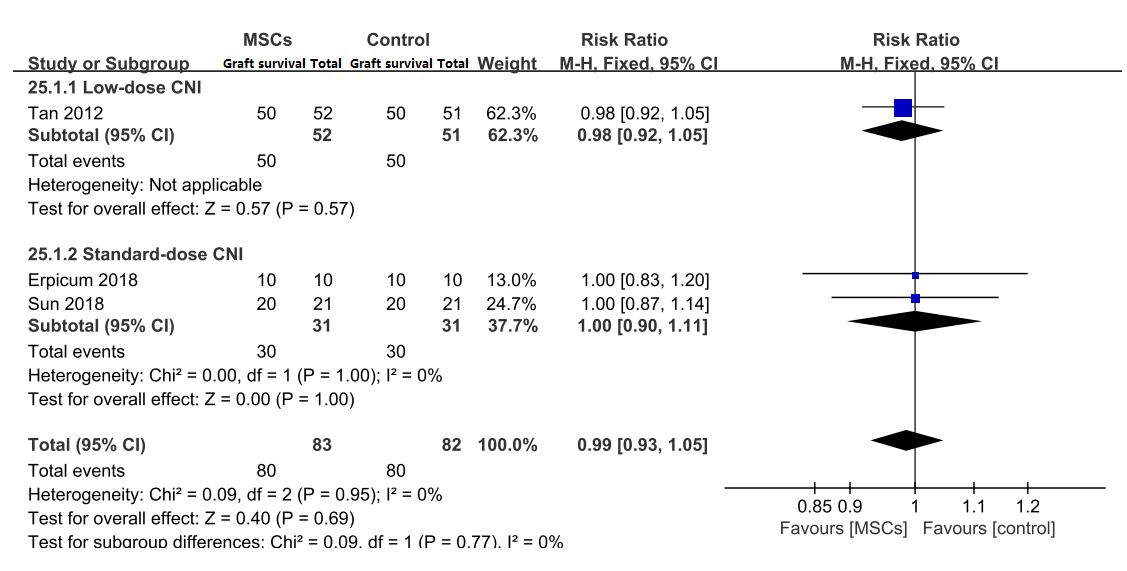

Supplement: Supplementary file 2 — Additional file 2: Supplementary Figure 2. Effect on 1-year graft survival rate between low-dose CNI group and the standard-dose CNI group. [file 13287_2021_2219_MOESM2_ESM.tif]

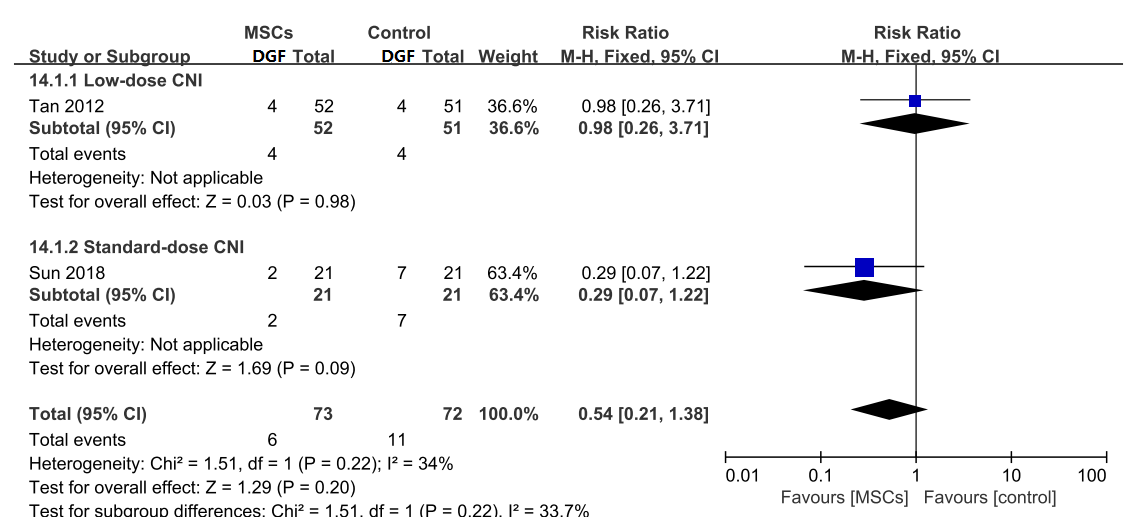

Supplement: Supplementary file 3 — Additional file 3: Supplementary Figure 3. Effect on 1-year infection between low-dose CNI group and the standard-dose CNI group. [file 13287_2021_2219_MOESM3_ESM.tif]

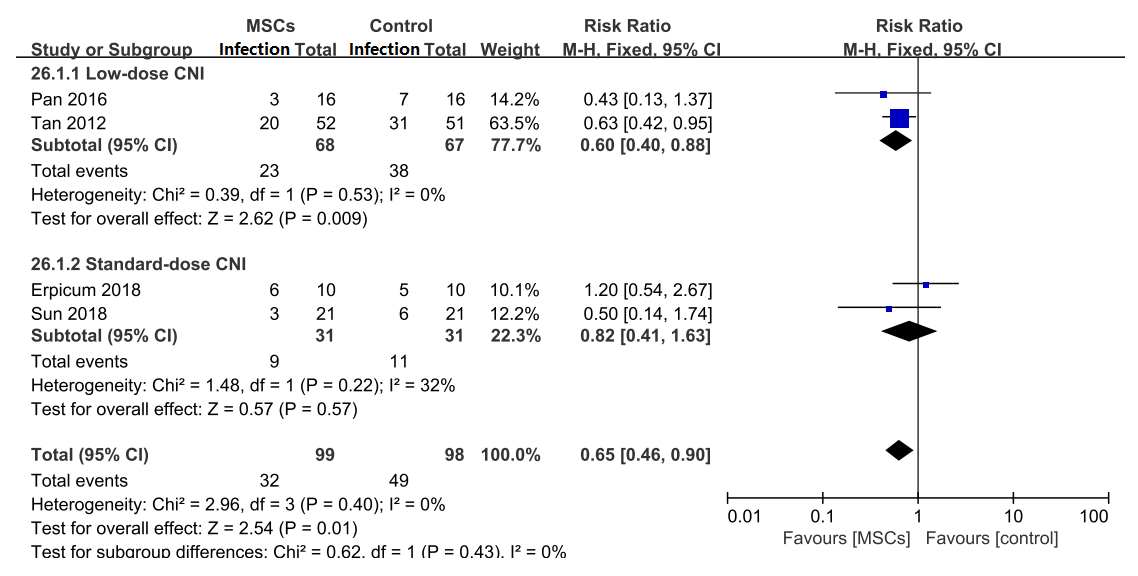

Supplement: Supplementary file 4 — Additional file 4: Supplementary Figure 4. Effect on DGF rate between low-dose CNI group and the standard-dose CNI group. [file 13287_2021_2219_MOESM4_ESM.tif]

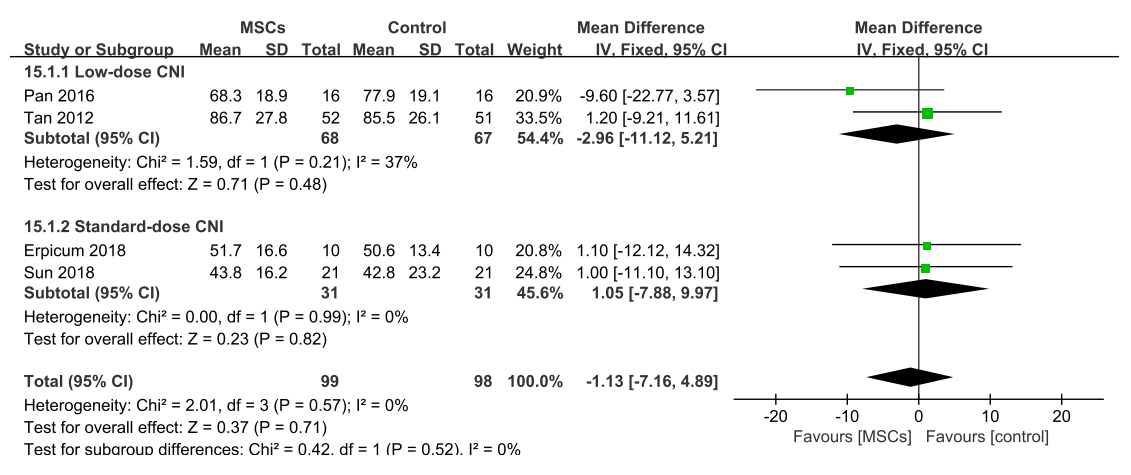

Supplement: Supplementary file 5 — Additional file 5: Supplementary Figure 5. Effect on renal graft function at 12 months post surgery between low-dose CNI group and the standard-dose CNI group. [file 13287_2021_2219_MOESM5_ESM.tif]

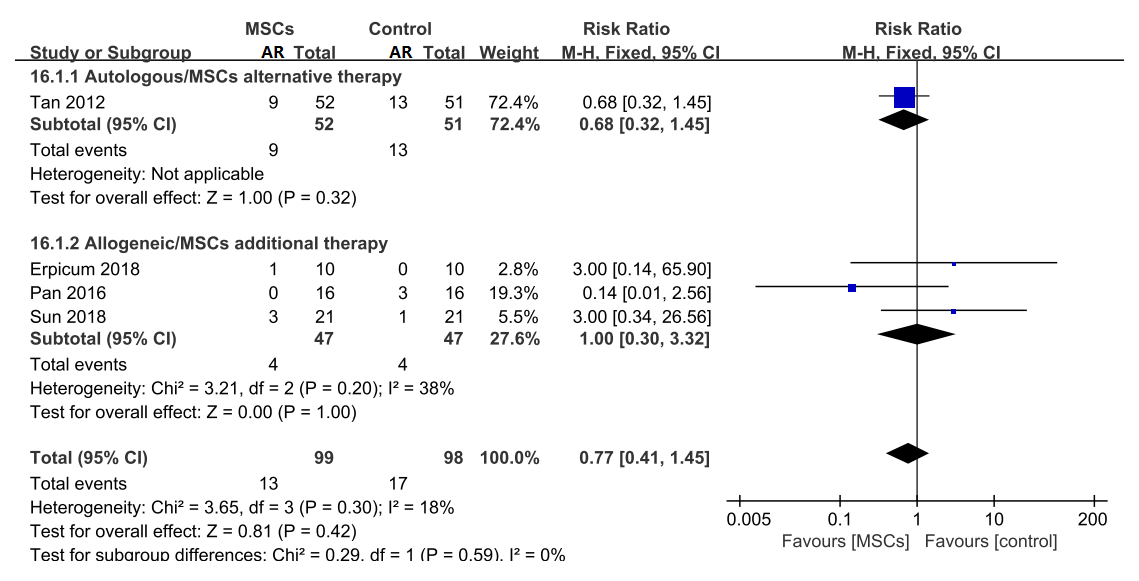

Supplement: Supplementary file 6 — Additional file 6: Supplementary Figure 6. Effect on 1-year AR rate between autologous/MSC alternative therapy group and allogeneic/MSC additional therapy group. [file 13287_2021_2219_MOESM6_ESM.tif]

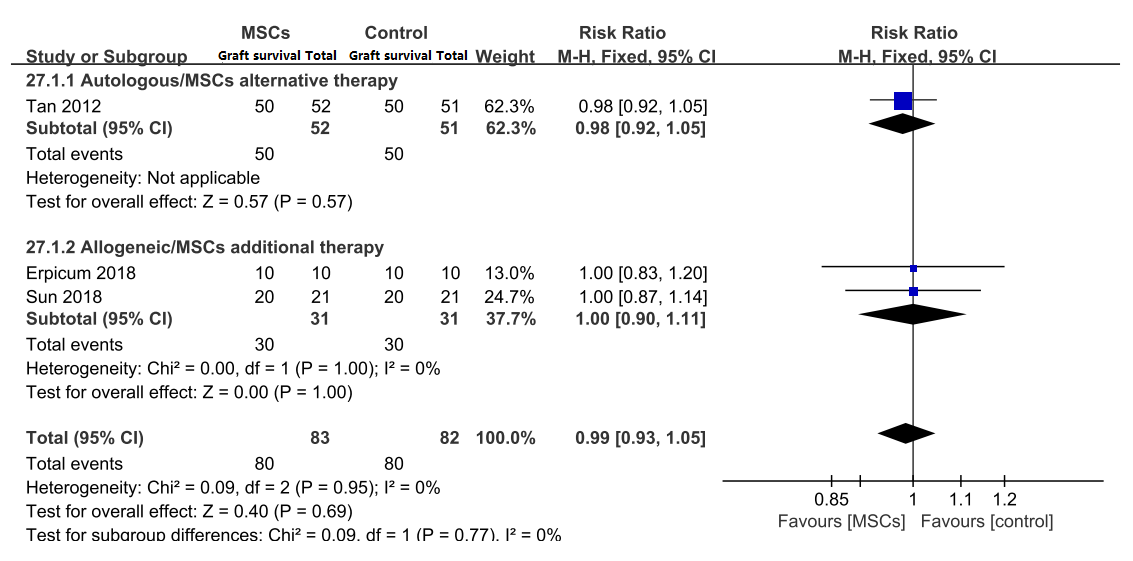

Supplement: Supplementary file 7 — Additional file 7: Supplementary Figure 7. Effect on 1-year graft survival rate between autologous/MSC alternative therapy group and allogeneic/MSC additional therapy group. [file 13287_2021_2219_MOESM7_ESM.tif]

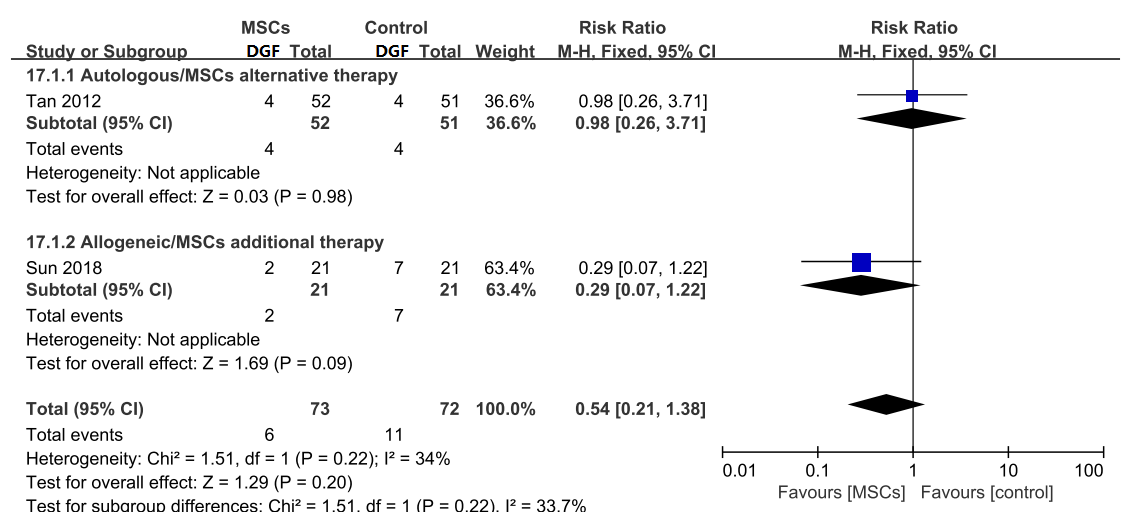

Supplement: Supplementary file 8 — Additional file 8: Supplementary Figure 8. Effect on 1-year infection between autologous/MSC alternative therapy group and allogeneic/MSC additional therapy group. [file 13287_2021_2219_MOESM8_ESM.tif]

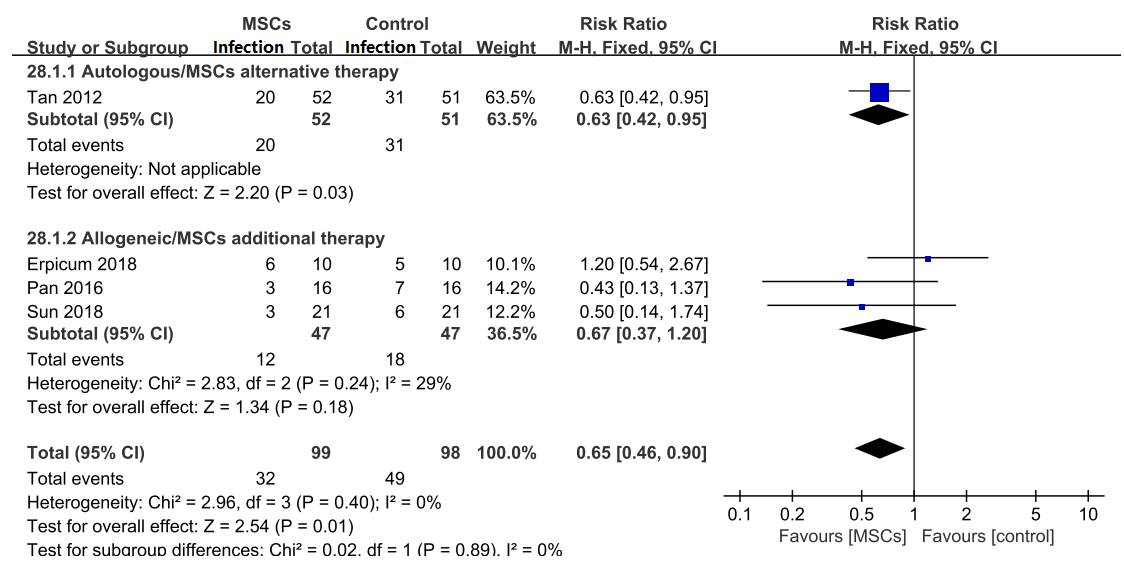

Supplement: Supplementary file 9 — Additional file 9: Supplementary Figure 9. Effect on DGF rate between autologous/MSC alternative therapy group and allogeneic/MSC additional therapy group. [file 13287_2021_2219_MOESM9_ESM.tif]

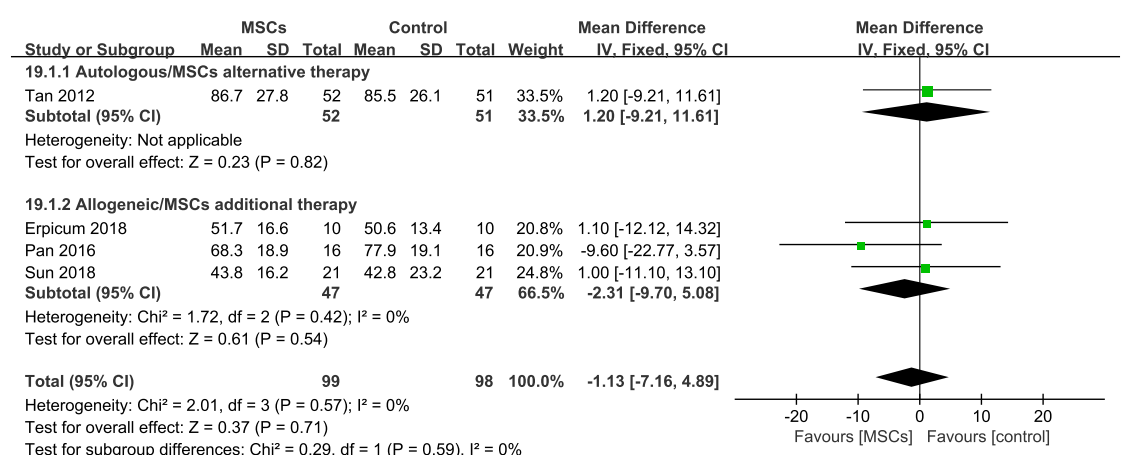

Supplement: Supplementary file 10 — Additional file 10: Supplementary Figure 10. Effect on renal graft function at 12 months post surgery between autologous/MSC alternative therapy group and allogeneic/MSC additional therapy group. [file 13287_2021_2219_MOESM10_ESM.tif]
